# 

**Published:** 1900-04-15

**Authors:** 


### ANNOUNCEMENTS.

The National Association of Dental Faculties will meet at Old Point Comfort, Va., the afternoon of July 13, 1900.

J. H. Kennerly, *Sec'y.*

---

The Missouri State Dental Association meets July 10th, 11th, 12th and 13th, at Louisiana, Mo.

B. L. Thorpe, *Cor. Sec'y,*
St. Louis, Mo.

---

The Tri-State Dental Association, of Indiana, Illinois and Kentucky, meets at Evansville, Ind., May 8th, 9th and 10th.

W. H. Brosman, *Sec'y,*
Albion, Ills.

---

The Northern Ohio Dental Association meets at Cleveland, Ohio, June 5th, 6th and 7th, at the Colonial Hotel.

The completed program is already in print and may be had by addressing Dr. W. T. Jockman, Cleveland, Ohio.

---

THE thirtieth annual meeting of the Kentucky State Dental Association will be held in Louisville, May 29th, and continue for three days. The program, which we have at hand, calls for twenty-six papers and twenty-one clinics. How this is all to be done in three short days we shall know after attending this meeting. No such array of distinguished professional writers and clinicians has appeared outside of our national meetings. We are sorry that the limits of our space will not permit the publication of the program.

---

THE Michigan State Board of Examiners in Dentistry will meet in Grand Rapids, May 8th, at 9 A. M. For particulars address Dr. F. O. GILBERT, *Sec'y*,
Bay City, Mich.

---

### NATIONAL DENTAL ASSOCIATION.

The meeting of the Association, which was to have been held June 26th at Old Point Comfort, has been changed by vote of the members to Tuesday, July 10th. Reports from officers of the sections indicate that we shall have a very high order of work done at this meeting. Men have been selected to write papers because of their peculiar fitness for the undertaking, and enough of those invited have accepted to insure a full program. A special feature of the meeting will be clinics, under the supervision of Section III. It is hoped the meeting may be as largely attended as that of last year at Niagara Falls.

B. HOLLY SMITH, *Pres.*

The New York State Dental Society will meet at Albany, N. Y., May 9th and 10th. Papers will be read by Drs. F. J. Capon, Joseph Head, E. C. Kirk, S. B. Palmer, Charles H. Barnes and Milton F. Smith.

W. A. White, D.D.S., *Sec'y.*

---

The Illinois State Board of Dental Examiners will meet in Chicago, May 3rd, 4th and 5th, at the Chicago Business College, 67 Wabash Avenue. All interested should correspond with Dr. J. H. Smyser, the secretary, 70 State Street, at least ten days previous to the date of meeting.

---

The Illinois State Dental Association will meet at Springfield, May 8th, 9th and 10th. A good program is provided.

*President's Address.*—Dr. R. N. Lawrence.

*Reports of Committees.*—Dr. Harlan, on "Dental Science and Literature;" Dr. Goslee, on "Dental Art and Invention."

*Papers.*—Dr. J. E. Nyman, "Gold Crown;" Dr. W. W. Moorhead, "Prosthetic Dentistry;" Dr. W. A. Johnson, "Treatment of Fractured Lower Jaw;" Dr. G. Monroe, "Calcification a Controlling Factor in the Treatment of Teeth;" G. W. Entsminger, "Habits Incident to the Dental Profession;" G. E. Lobb, "Electricity and its Value in Dentistry;" Dr. A. H. Peck, "Pyorrhea Alveolaris, So-Called;" Dr. D. M. Cattell, "To Emphasize Some Things in Operative Procedures;" Dr. Edmund Noyes, "A Subject Connected with Operative Dentistry;" Dr. E. Malohinny, "Antiseptics;" Dr. J. E. Hinkins, "Report of Clinics."

*Clinics.*—Dr. C. W. Gilmer, Electro-Plating Apparatus; Dr. P. A. Pyper, Platinum and Gold Contour Filling; Dr.

J. O. Brown, Contour Filling with Watt's Crystal Gold; J. N. McDowell, Orthodontia Models and Regulating Appliances; Dr. P. E. Roach, Rubber Teeth in Crown and Bridge Work; Dr. W. W. Tobey, Contour Filling; Dr. A. J. Elmer, Pyorrhea Alveolaris; Dr. T. W. Brophy, Surgical Clinic; Dr. W. F. Green, Root-Canal Filling; Dr. Geo. W. Cook, Surgical Treatment of Pyorrhea; Dr. L. H. Stewart, Continuous Gum; Dr. J. E. Nyman, Carving Solid Gold Cusp; Dr. W. V. B. Ames, Cements Under the Microscope; Dr. J. R. Rayburn, Gold Filling with Powers' Engine Mallet; Dr. M. L. Hannaford, Distal Cuspid Gold Filling; Dr. G. A. Miller, Seamless Gold Crown; Dr. W. F. Fowler, Porcelain Inlay; Dr. R. Goode, Pyorrhea Alveolaris; Dr. A. F. James, Immediate Regulating; Dr. C. N. Thompson, Porcelain Inlay; Dr. L. S. Tenny, Gold Restoration of Anterior Tooth; Dr. F. V. Yorker, Gold Filling; Dr. L. W. Nevins, Extracting with Nitrous Oxide.

---

THE next meeting of the American Medical Association will be held at Atlantic City, June 5 to 8, 1900. The Section on Stomatology presents the following program:

SYMPOSIUM ON DENTAL EDUCATION.

1. Relations of Dental and Oral Surgery to General Medicine—Professional Status of Properly Educated Practitioners of Dental and Oral Surgery, Dr. N. S. Davis, Sr.; 2. Preliminary Qualifications, Dr. J. Taft; 3. Course of Study, Dr. W. A. Evans; 4. Methods of Teaching (didactic or recitational), Dr. A. H. Peck; 5. Shall the Dental Student be Educated Independently of General Medicine? Dr. G. V. I. Brown; 6. Is Medical Education a Necessary Qualification for Dental Practice? Drs. Alice Steeves and R. R. Andrews; 7. The Practical Value of a Medical Education in Dental Practice, Dr. W. B. Hill; 8. Technical Training vs. Theoretic, Dr. John S. Marshall; 9. Should the Medical Undergraduate be Instructed in the Principles of Dentistry? Dr. M. L. Rhein; 10. Post-graduate Study in Dentistry and Degrees Therefor, Dr. W. E. Walker; 11. Handwriting upon the Wall—What Does it Portray? Dr. A. E. Baldwin; 12. Limitations, Dr. Eugene S. Talbot.

SYMPOSIUM ON INTERSTITIAL GINGIVITIS, OR SO-CALLED PYORRHEA ALVEOLARIS.

1. Etiology, Dr. G. Lenox Curtis; 2. Neurotic Affections, Dr. J. G. Kiernan; 3. Indigestion Auto-intoxication, Dr. Eugene S. Talbot; 4. Chemical Factors in Etiology, Dr. W. L. Baum; 5. Constitutional Treatment, Dr. J. H. Salisbury; 6. Local Treatment, Dr. M. H. Fletcher.

PROGRAM CONTINUED.

1. So-called Glands in the Peridental Membrane, Dr. M. H. Fletcher; 2. The Evolution of Decay, continued, Dr. Arch. C. Hart; 3. Co-operation of the Public Schools in Teaching; Good Teeth; Good Health; whatever we wish to see introduced into the life of a nation must be introduced into its schools, Dr. Richard Grady; 4. Subject to be announced, Dr. V. A. Latham.

The Section on Stomatology will meet at Hotel Senate. The officers of the section invite all to be present and to take part in the discussions.

Those who wish to join the Association must obtain credentials from their State or local dental societies and the payment of $5 to the Secretary of the Association. This will entitle them to the Journal for one year.

Accommodation can be had by writing F. B. Cook & Son, Hotel Senate.

EUGENE S. TALBOT,
*Sec'y Section on Stomatology.*

